# The Evolution of Molybdenum Dependent Nitrogenase in Cyanobacteria

**DOI:** 10.3390/biology10040329

**Published:** 2021-04-14

**Authors:** Tomoaki Watanabe, Tokumasa Horiike

**Affiliations:** 1 United Graduate School of Agricultural Science, Gifu University, Gifu 501-1193, Japan; z6102003@edu.gifu-u.ac.jp; 2 Department of Bioresource Sciences, Shizuoka University, Shizuoka 422-8529, Japan

**Keywords:** nitrogen fixation-related genes, nitrogen fixation, cyanobacteria, horizontal gene transfer, evolution

## Abstract

**Simple Summary:**

Nitrogen fixation is the process by which nitrogen in the atmosphere is converted into ammonia and other nitrogen-containing organic compounds. It is carried out by a variety of bacteria, including Cyanobacteria. Previous studies have shown that several groups of Cyanobacteria have the ability to fix nitrogen; however, because these groups are scattered throughout the Cyanobacterial lineage, the evolutionary history of nitrogen fixation in these bacteria has not been clarified. In this study, we attempted to identify the origin of nitrogen fixation development in Cyanobacterium by focusing on molybdenum dependent nitrogenase, a major nitrogen fixing enzyme. We compared a phylogenetic tree from 179 species of Cyanobacteria to one generated from nitrogen fixation-related genes. We also compared the genomic locations of those genes. As a result, we found that nitrogen fixing genes were acquired in the Cyanobacterium common ancestor and subsequently lost in some lineages. The results demonstrate that inconsistencies between species phylogeny and organism characteristics can occur and be caused not only by horizontal gene transfer, but also by gene deletion.

**Abstract:**

Nitrogen fixation plays a crucial role in the nitrogen cycle by helping to convert nitrogen into a form usable by other organisms. Bacteria capable of fixing nitrogen are found in six phyla including Cyanobacteria. Molybdenum dependent nitrogenase (*nif*) genes are thought to share a single origin as they have homologs in various phyla. However, diazotrophic bacteria have a mosaic distribution within the cyanobacterial lineage. Therefore, the aim of this study was to determine the cause of this mosaic distribution. We identified *nif* gene operon structures in the genomes of 85 of the 179 cyanobacterial strains for which whole genome sequences were available. Four *nif* operons were conserved in each diazotroph Cyanobacterium, although there were some gene translocations and insertions. Phylogenetic inference of these genes did not reveal horizontal gene transfer from outside the phylum Cyanobacteria. These results support the hypothesis that the mosaic distribution of diazotrophic bacteria in the cyanobacterial lineage is the result of the independent loss of *nif* genes inherited from common cyanobacterial ancestors in each lineage.

## 1. Introduction

Nitrogen fixation is the biochemical process by which the atmospheric nitrogen is converted to nitrogen-containing compounds including assimilation into organic compounds as part of the nitrogen cycle. Diazotrophic species are present in multiple phyla of bacteria and archaea [1]. Genes encoding nitrogen fixation enzymes are categorized into five groups (I–V) [2]. The genes related to nitrogen fixation such as *nifH* and *nifD* are conserved among species in different phyla of both bacteria and archaea [2,3]. Although some *nif* operons have been lost, duplicated, or horizontally transferred [4,5], many are conserved among these phyla [2]. These results indicate that *nif* genes originated from a common ancestor [6].

However, in the phylum Cyanobacteria, diazotrophic species are distributed in a mosaic pattern. Two explanations for this have been provided. One is that the last common ancestor of the Cyanobacteria did not have the ability to fix nitrogen, and some lineages independently obtained this ability by horizontal gene transfer from other phyla [7,8,9,10]. Alternatively, the last common ancestor may have been capable of nitrogen fixation, but this ability was subsequently lost in some lineages [11,12]. The former hypothesis is supported by evidence from a small number of species; however, diazotrophic species that diverged from the common ancestor of Cyanobacteria were not included in these analyses [7,8,9,10]. For example, *Synechococcus* sp. JA-3-3Ab, which can fix nitrogen and diverged earlier, was not included in these analyses [12]. Therefore, the former hypothesis is not well supported by the currently available data. In contrast, the studies that supported the latter possibility did not consider the possibility of horizontal gene transfer. As there are many horizontally transferred genes in Cyanobacterial genomes [13], this possibility cannot be ignored. Therefore, a phylogenetic analysis of each *nif* gene in Cyanobacteria was conducted to verify the potential of each hypothesis.

In the cyanobacterial genome, the *nif* genes form operon structures such as *nifBSU, nifHDK*, *nifENXW*, and *nifVZT* [14]. In this study, we identified the operon structures of the *nif* genes in each genome and performed a phylogenetic analysis of the proteins encoded by each operon structure to examine the reasons for the mosaic distribution of diazotrophic Cyanobacteria.

## 2. Materials and Methods

### 2.1. Construction of a Phylogenetic Tree of 179 Cyanobacteria Species

Genomic sequence data for 179 Cyanobacteria (Table S1) and five outgroups (*Bacillus subtilis* 168, *Clostridium botulinum* A str. ATCC 3502, *Mycobacterium tuberculosis* CDC1551, *Streptomyces coelicolor* A3 (2), and *Escherichia coli* K-12 MG1655) were obtained in the GenBank format from the National Center for Biotechnology Information database (ftp://ftp.ncbi.nlm.nih.gov/genomes/genbank/bacteria/, 13 September 2020). Of the 179 species, 85 belong to a diazotroph genus (Table S1). We detected 31 marker proteins for species phylogenetic analysis using AMPHORA2 [15]. Each species’ marker protein sequences were concatenated into one sequence. Multiple alignments of the concatenated sequences were performed using MAFFT version 7.271 [16]. Using the alignment data, a species phylogenetic tree was constructed based on the maximum likelihood method using RaxML version 8.29 [17]. Bootstrap tests were performed with the “-# autoMR” option. The optimal substitution model was estimated using RaxML.

### 2.2. Collection of nif Amino Acid Sequence Data

First, OrthoFinder [18] was used to generate ortholog datasets for all proteins. In these datasets, we detected proteins annotated with “nif’” and identified their orthologs. All proteins belonging to orthogroups [18] were considered nif candidates. For example, if a protein belonged to an orthogroup containing nifD, it was considered a nifD candidate. There was a *nifD* gene in *Nostoc punctiforme* PCC 73102 that was not registered by the coding sequence, we manually added information about its encoded protein. *NifE* and *nifN* genes are fused in some genomes (*Anabaena variabilis* ATCC 29413, *Anabaena* sp. YBS01, *Calothrix* sp. NIES-2098, *Calothrix* sp. NIES-2100, *Trichormus variabilis* 0441, *Calothrix brevissima* NIES-22, *Chroococcidiopsis thermalis*, *Thermoleptolyngbya* sp. PKUAC-SCTA183, and *Trichodesmium erythraeum* IMS101). The fused nifs are called nifEN.

### 2.3. Detection of nif Gene Operon Structures

Location information for each *nif* gene in each genome was obtained from the GenBank file. Based on this information, *nif* genes with the potential to form known *nif* operons (*nifBSU, nifENXW, nifHDK, nifVZT*) were detected, and if there were <4 genes between neighboring *nif* genes, they were considered to be in the same operon. For example, if *nifB*, *nifS*, and *nifU* were in close proximity, they were considered to form one operon. In addition, if ≥4 genes existed between *nif* genes but a *nif* operon was present upstream and downstream of the *nif* gene, these genes were considered part of the same operon. In some genomes (for example, *A. variabilis* ATCC 29413 and *Leptolyngbya boryana*), the *nif* genes comprise of one large operon such as *nifBSUHDKENXW* [19,20]. However, in this analysis, this operon was treated as separated operons (*nifBSU*, *nifHDK*, and *nifENXW*) for comparison with other *nif* operons.

### 2.4. Selection of nif Genes from Multiple nif Genes in An Operon Structure

If there were multiple *nif* genes believed to be in the operon structure (for example, two *nifB* genes in an operon), we chose one coding sequence for each *nif* gene as follows. First, a phylogenetic tree was inferred using all nif proteins encoded by the *nif* genes in the operon by the maximum likelihood method using RaxML. These phylogenetic trees were compared to the species phylogenetic tree, and the nif whose phylogenetic position was more similar to the species phylogenetic tree was selected. The chosen nifs and the *nif* genes were used for later analyses.

### 2.5. Phylogenetic Analysis of Cyanobacterial nif Proteins

Proteins used in the phylogenetic analysis were nifB, nifD, nifE, nifH, nifK, nifN, nifS, nifT, nifU, nifV, nifW, nifX, and nifZ. Of the 179 cyanobacterial species, 82–85 species (depending on the type of *nif*) contained nifs that met the above criteria. Therefore, we performed phylogenetic analysis of the nifs in these species. Using the alignment data, phylogenetic trees were constructed in RaxML using the maximum likelihood method. Bootstrap tests were performed with the “-# autoMR” option. The optimal substitution model was estimated using RaxML. All other parameters were set to default values.

### 2.6. Discovery and Phylogenetic Analysis of nifs in Non-Diazotrophic Cyanobacteria

All ortholog sequences for nifs encoded by the *nif* genes comprising the operons estimated in Section 2.3 were obtained from non-diazotrophic species. Phylogenetic analysis was performed on the nif proteins of non-diazotrophs and diazotrophs as described in Section 2.5.

## 3. Results and Discussion

### 3.1. Phylogenetic Tree of 179 Cyanobacteria Species

**Figure 1:**
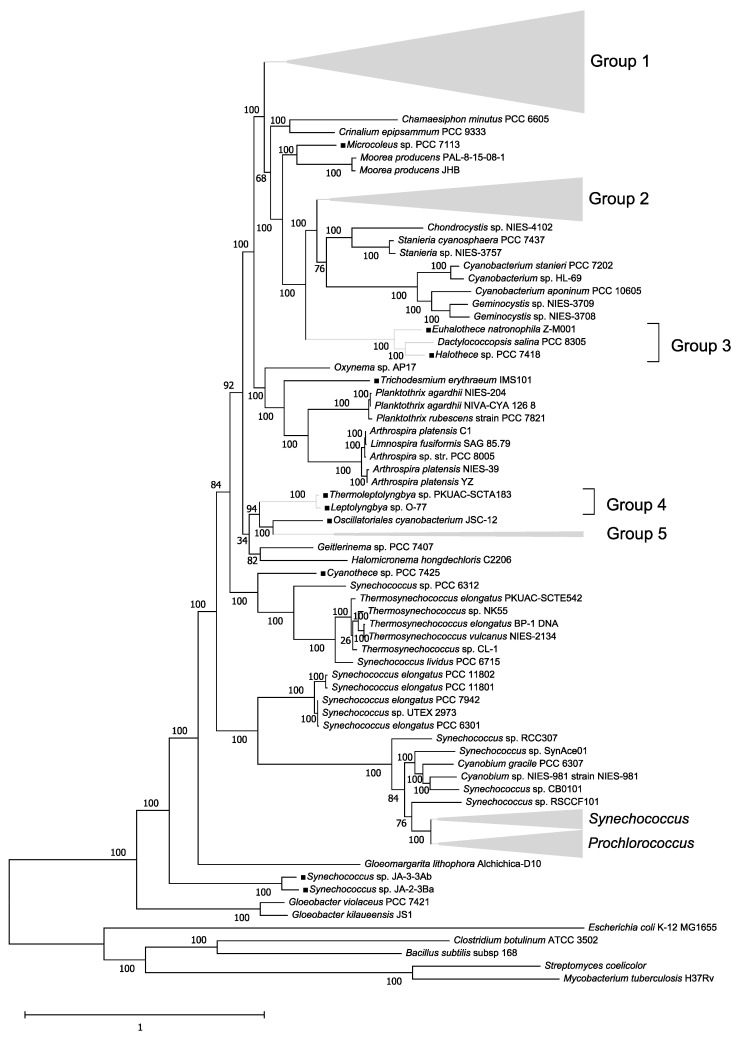
Phylogenetic tree of 179 Cyanobacteria species. The phylogenetic tree was inferred using the concatenated sequences. Some of the phylogenetic relationships among species are compressed in Figure 1, but the complete phylogenetic tree is shown in Figure S1. Black squares indicate diazotrophic Cyanobacteria. Diazotrophic Cyanobacteria belonging to groups I–V are shown in brackets. Diazotrophic cyanobacteria with no closely related species were not included in the groups. Bootstrap values are shown at the internal branches (<100).

A phylogenetic tree of the 179 concatenated Cyanobacteria genomes was constructed (Figure 1). Proteobacteria (*E. coli*), Actinobacteria (*M. tuberculosis* and *S. coelicolor*), and Firmicutes (*B. subtilis* and *C. botulinum*) species were used as outgroups. The first species to diverge from the common ancestor of Cyanobacteria belonged to the genus *Gloeobacter*. This genus consists of primitive Cyanobacteria in which a portion of the genes related to the thylakoid membranes and photochemical systems I and II are not present, and it has been previously suggested to be the first branch from the common ancestor of Cyanobacteria [21]. As many Cyanobacteria have synonyms, Cyanobacteria belonging to one genus may not be monophyletic; however, species belonging to the same genera formed roughly the same clusters.

### 3.2. Operon Structures of the nif Genes

**Figure 2:**
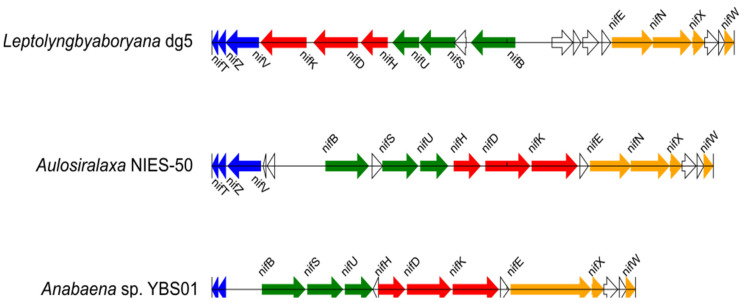
Operon structures of Cyanobacteria *nif* genes. The *nif* operon structures of *Leptolyngbya boryana* dg5, *Aulosira laxa* NIES-50, and *Anabaena* sp. YBS01 are shown. Blue, red, green, and orange arrows indicate the *nifVZT, nifHDK*, *nifBSU*, and *nifENXW* operons, respectively. White arrows indicate the genes unrelated to nitrogen fixation.

Of the 179 Cyanobacteria, *nif* genes were identified in the genomes of the 85 species thought to be diazotrophs including those comprising the *nifBSU*, *nifHDK*, *nifENXW*, and *nifVZT* operons. The operon structures of representative cyanobacterial *nif* genes are shown in Figure 2. A map of *nif* genes comprising operon structures in the cyanobacterial genome is shown in Table S2. There were some translocations and insertions in places, but each operon was essentially conserved.

In nine Cyanobacteria (*Anabaena* sp. YBS01, *A. variabilis* ATCC 29413, *A. laxa* NIES-50, *C. brevissima* NIES-22, *Calothrix* sp. NIES-2098, *Calothrix* sp. NIES-2100, *T. tenuis* PCC 7101, *T. variabilis* 0441, and *N. carneum* NIES-2107), two *nif* operon sets were present at distant positions from each other in the genome. The *nifVZT* operon was more conserved than the other *nif* operons, and no other genes were present among the three *nif* genes. In rare cases, only two genes (*nifV* and *nifZ*, *nifZ* and *nifT*, or *nifV* and *nifT)* formed an operon; in these cases, the other *nif* gene was located at a distance (Table S2). Genes in the *nifHDK* operon encode a protein that plays a central role in nitrogen fixation, but the operon structure is not highly conserved (Table S2). Nevertheless, *nifH*, *nifD*, and *nifK* did not often exist on their own, and many species maintained an operon structure for at least two genes (either *nifH* and *nifD* or *nifD* and *nifK*). There were also several species in which the *nifK* gene was found upstream of *nifENXW* and was considered a part of the same operon. Several species were identified in which multiple genes were inserted between *nifD* and *nifK*, which would have presumably originally formed an operon. The *nifB-fdxN-nifSU* operon typically assumed its basic form; however, there were often a few genes between *nifB* and *nifS* including *fdxN*. *nifS* and *nifU* were always adjacent to each other, while *nifB* sometimes formed operons at a distance to *nifSU* or did not form operons. Cyanobacterium endosymbiont of *Epithemia turgida* isolate EtSB Lake Yunoko lacked a *nifU* gene; however, these contained a pseudogene for *nifU* downstream of *nifS*. This *nifU* gene was remotely located, did not form an operon, and had low sequence similarity, suggesting that it serves as an alternative to the original *nifU* gene. The *nifENXW* operon was relatively well conserved. Most Cyanobacteria formed an operon structure with *nifE*, *nifN*, and *nifX* adjacent to each other and *nifW* between genes of unknown function.

### 3.3. Phylogenetic Analysis of nif Proteins

We inferred phylogenetic trees for each nif encoded by a *nif* gene contained in an operon structure (Figure S1a–m). The common ancestor of Synechococcus sp. JA-3-3 Ab and Synechococcus sp. JA-2-3 Ba diverged first among the diazotrophic Cyanobacteria in the species phylogenetic tree (Figure 1), therefore, we used these species as the outgroup in each tree. If nifs are true orthologs, the tree shapes should be consistent. We divided the diazotrophic Cyanobacteria into five groups (note that this classification is not the traditional classification of Cyanobacteria, section I–V [22]) based on the species phylogenetic trees to compare phylogenetic trees easily (Table S1). However, the two species used as an outgroup and five diazotrophs with no related species (C. thermalis PCC 7203, Microcoleus sp. PCC 7113, T. erythraeum IMS 101, Cyanothece sp. PCC 7425, and Oscillatoriales cyanobacterium JSC-12) were not included in these groups. Groups I–V were all monophyletic (Figure 1). The following discussion is based on the positions of these groups within the phylogenetic trees.

As above-mentioned, nine Cyanobacteria had two sets of *nif* operons. A previous study reported the presence of two sets of *nif* operons (*nif1* and *nif2*) in the genome of A. variabilis ATCC 29413 [23]. In this study, of the two sets of nifs, we considered the nif closely related to nif1 of A. variabilis ATCC 29413 on the nif phylogenetic tree to be nif1, and the nif closely related to nif2 to be nif2 (Figure 1). *Nif1* genes have been inherited from the common ancestor of diazotrophic Cyanobacteria because the locations of nif1 on the nif phylogenetic tree are similar to those on the species phylogenetic tree. However, *nif2* genes may have arisen before the common ancestor of group I diverged from the other groups by horizontal gene transfer. All Cyanobacteria with nif2 form heterocysts. It is possible that A. variabilis ATCC 29413 was able to fix nitrogen efficiently by using both the original nif1 and the nif2 coded by *nif2* genes introduced by horizontal transfer [23].

The phylogenetic tree of nifB, encoded by the *nifBSU* operon, resulted in monophyly for all groups, whereas the nifS and nifU trees did not (Figure S1a–c). In the nifS tree, group III was contained within group II; and in the nifU tree, groups II and III were contained in group I. All three phylogenetic trees for the proteins encoded by the *nifHDK* operon were monophyletic (Figure S1d–f). In particular, the phylogenetic positions of the groups in the nifD and nifK trees were almost identical, suggesting that they evolved in the same way. The three phylogenetic trees for proteins encoded by the *nifVZT* operon were different (Figure S1g–i). The nifV tree was monophyletic for all groups. The nifZ phylogenetic tree included all groups except for group I in group I. In the nifT tree, group I’ was divided into two, one of which included group IV. Of the four phylogenetic trees for nif proteins encoded by the *nifENXW* operon, only the nifE tree was monophyletic for all groups (Figure S1j–m). The nifN tree included group III in group II, the nifX tree included group IV in group I’, and group I in the nifW tree was paraphyly.

The phylogenetic relationships within each group were similar for the *nifBSU*, *nifHDK*, *nifVZT*, and *nifENXW* operons, but the branching patterns between some groups were not consistent. The branches that differed among phylogenetic trees corresponded to branches with low bootstrap values. Such branches are thought to be unreliable. However, some nifs (nifX, nifW, nifZ, and nifT) had many internal branches with low bootstrap values, and these nifs showed larger differences in phylogenetic tree shape from other nifs coded by the same operon. The average amino acid length of nifX, nifW, nifZ, and nifT was 136.8, 106.4, 93.6, and 66.9, respectively. The short amino acid length of these nifs may have prevented accurate phylogenetic inference due to their small amount of information, resulting in many unreliable internal branches.

None of the nifs encoded by the operons had long external branches, suggesting that horizontal gene transfers from outside the phylum Cyanobacteria did not occur during the evolution of the Cyanobacterial *nif* genes. In contrast, a previous study has reported the horizontal transfer of *nif* genes from Deltaproteobacteria [24].

### 3.4. Nif Genes in Non-Diazotrophic Cyanobacteria

Orthologs of nif were detected in four non-diazotrophic Cyanobacteria (Table 1): nifS in *Cyanobacterium aponinum* PCC 10605 and *Cyanobacterium stanieri* PCC 7202, and nifV in *Moorea producens* PAL-8-15-08-1 and *M. producens* JHB. The *nif* genes encoding these proteins could have been derived from a common Cyanobacteria ancestor or through horizontal transfer from different phyla. If the former is true, the species may have originally been diazotrophs; however, after losing their nitrogen-fixing ability, their *nif* genes were gradually lost. However, some of these genes are thought to have functions other than nitrogen-fixing and were not lost. In this case, as the remaining *nif* genes are orthologous to those of closely related diazotrophs, the positions of the species on the nif phylogenetic trees would be expected to be the same or similar to their positions on the species phylogenetic tree. Conversely, if the latter is true, the nifs of non-diazotrophs and diazotrophs that are phylogenetically distant to each other could be closely related in the nif phylogenetic trees.

According to the phylogenetic tree, *C. aponinum* PCC 10605 and *C. stanieri* PCC 7202 were closely related, and both are non-diazotrophs that contain *nifS* genes (Figure 1). However, in the nifS tree, the internal branches leading to these two species diverged from near the roots of the phylum and were extremely long (Figure S2a). Therefore, the *nifS* genes of the ancestor of these species may have resulted from horizontal transfer from outside the phylum Cyanobacteria. In the species phylogenetic tree, *M. producens* PAL-8-15-08-1 and *M. producens* JHB were closely related (Figure 1). Conversely, in the nifV phylogenetic tree, the external branches connected to these species were very long (Figure S2b). Thus, the *nifV* genes in these species are likely to have been independently obtained through horizontal transfer from outside the phylum Cyanobacteria.

### 3.5. General Discussion

The phylogenetic trees of the nifs coded by the *nif* operon (Figure S1) did not have very long branches, like those leading to the nifs of non-diazotrophic Cyanobacteria (Figure S2). This suggests that after the common ancestor of diazotrophic Cyanobacteria acquired the first *nif* operon, none of the *nif* genes comprising the operon were acquired by horizontal transfer from outside the phylum Cyanobacteria. The fact that the *nif* operon is conserved also supports that horizontal gene transfer is restricted. As a result, it is conceivable that the *nif* genes of each lineage of diazotrophic Cyanobacteria were derived from the *nif* genes of the common ancestor of diazotrophic Cyanobacteria, and that independent gene loss may have occurred in each lineage of the non-diazotrophic Cyanobacteria.

The topologies of nif phylogenetic trees of each *nif* operon were inconsistent, but the approximate phylogenetic relationships within and between groups were similar. In addition, the bootstrap values tended to be very low in the internal branches, where the phylogenetic relationships among the groups differed within the same operon. Therefore, it is possible that some errors occurred in phylogenetic inference among some groups.

In this analysis, we used 179 cyanobacterial species including 85 diazotrophs. As the number of species and lineages used was increased from the previous studies [7,8,9,10,11,12,14], it is presumed that reliable results were obtained. However, as a sufficient number of *nif* genes could not be detected, certain diazotrophic cyanobacteria were excluded from the analysis and therefore, could not be considered in this study. In future, we expect to explore other nitrogenases such as vnf and anf in diazotrophs that were excluded from this study; this would allow for a comprehensive understanding of the evolution of the nitrogen-fixing capacity of Cyanobacteria.

## 4. Conclusions

Phylogenetic analysis of 179 cyanobacterial species with sequenced genomes showed that diazotrophs are distributed in a mosaic pattern. We estimated the operon structures of 85 diazotroph species and found that all were conserved; however, there were some differences among many species. Phylogenetic trees of species and individual nif proteins were generated and compared, and many differences were found. The relatively conserved group structure and preserved operon structure suggest that horizontal transfer of the genes comprising the operons is restricted. Therefore, differences in the shape of the phylogenetic trees are more likely due to low accuracy in phylogenetic inference than horizontal gene transfer among Cyanobacteria.

Two sets of *nif* operons were detected in nine diazotrophic Cyanobacteria. One operon set is “*nif1* operon,” inherited from the common ancestor of diazotrophic Cyanobacteria with species divergence, and the other is “*nif2* operon,” inherited by horizontal gene transfer from within the phylum Cyanobacteria.

By comparing the phylogenetic tree of each nif protein, species phylogenetic tree, and the inferred operon structure, we were able to test the possibility of horizontal gene transfer within and outside Cyanobacteria. This approach will allow us to understand the origin of not only the nitrogen-fixing capacity of Cyanobacteria, but also the other properties of its mosaic distribution in the phylogenetic tree. We concluded that the mosaic distribution of diazotrophic bacteria in the cyanobacterial lineage is the result of the independent loss of *nif* genes inherited from common cyanobacterial ancestors in each lineage.

## Figures and Tables

**Table 1:** Non-diazotrophic Cyanobacteria with homologs to nifs coded by *nif* operons of diazotrophs.

Species	Nif
*Moorea producens* PAL-8-15-08-1	nifV
*Moorea producens* JHB	nifV
*Cyanobacterium stanieri* PCC 7202	nifS
*Cyanobacterium aponinum* PCC 10605	nifS

## Supplementary Materials

The following are available online at https://www.mdpi.com/article/10.3390/biology10040329/s1, Figure S1: Phylogenetic tree of 179 Cyanobacteria species. The phylogenetic tree was inferred using the concatenated sequences. Some of the phylogenetic relationships among species are compressed in Figure 1, but the complete phylogenetic tree is shown in Figure S1. Black squares indicate diazotrophic Cyanobacteria. Diazotrophic Cyanobacteria belonging to groups I–V are shown in brackets. Diazotrophic cyanobacteria with no closely related species were not included in the groups. Bootstrap values are shown at the internal branches (<100). Figure S2: Nif phylogenetic trees for diazotrophic Cyanobacteria. Phylogenetic trees of nifB (a), nifS (b), nifU (c), nifH (d), nifD (e), nifK (f), nifV (g), nifZ (h), nifT (i), nifE (j), nifN (k), nifX (l), and nifW (m) are shown. Diazotrophic Cyanobacteria belonging to groups I–V and I’ are shown in brackets. The nif1 are shown in black circles, whereas the nif2 are shown in black squares. Bootstrap values are shown at the internal branches (<100). Figure S3: Phylogenetic tree of nifV, which is coded by *nif* operon, and nifV, which is not. Diazotrophic Cyanobacteria belonging to groups I–V and I’ are shown in brackets. Black diamonds indicate the nifs that are not coded by the *nif* operons. Bootstrap values are shown at the internal branches (<100). Table S1: List of the Cyanobacteria species included in the analysis species with and without nitrogen fixation ability by “+”. Diazotrophic Cyanobacteria that have been grouped are indicated by their group numbers; those without groups are indicated by “None.” Table S2: Map of *nif* genes comprising the operon structure in the Cyanobacterial genome. The locations of all *nif* genes in each genome are shown. Each line indicates each genome. H, D, and K that are shown in red stand for *nifH*, *nifD*, and *nifK*, respectively. B, S, and U that are shown in green stand for *nifB*, *nifS*, and *nifU*, respectively. E, N, X, and W that are shown in brown stand for *nif E*, *nif N*, *nif X*, and *nif W*, respectively. V, Z, and T that are shown in blue stand for *nifV*, *nifZ*, and *nifT*, respectively. The numbers in white cells indicate the number of non-*nif* genes present.
