# Supplementary figures and images for: The Evolution of Molybdenum Dependent Nitrogenase in Cyanobacteria
Source: Biology (Basel). 2021 Apr 14;10(4):329. doi: 10.3390/biology10040329 (PMC8071049; doi:10.3390/biology10040329)

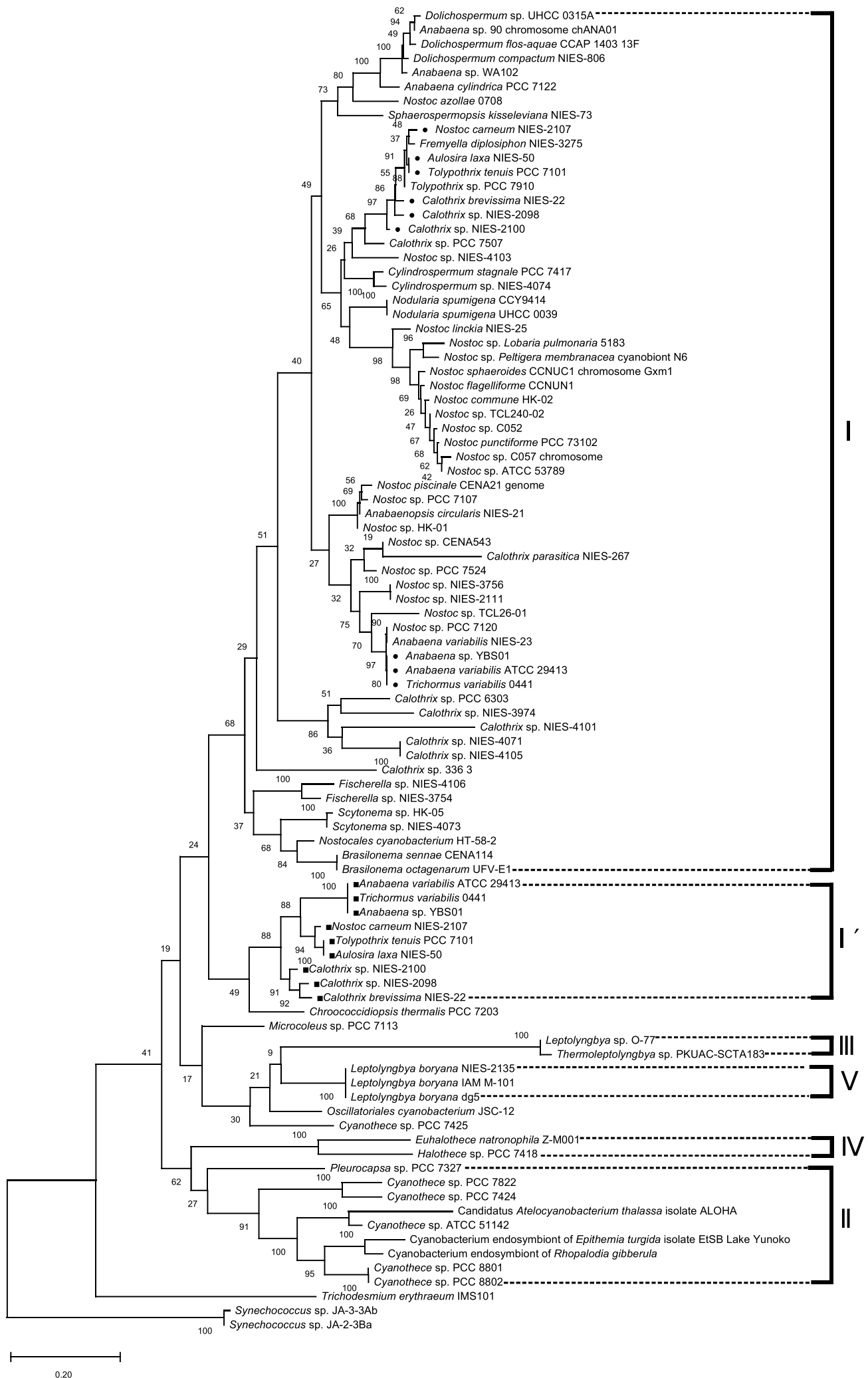

Figure S2a Phylogenetic tree of *nifB*

Supplement: Supplementary file 1 [file biology-10-00329-s001.zip › Figure S2a-2m/FigureS2a.pdf]

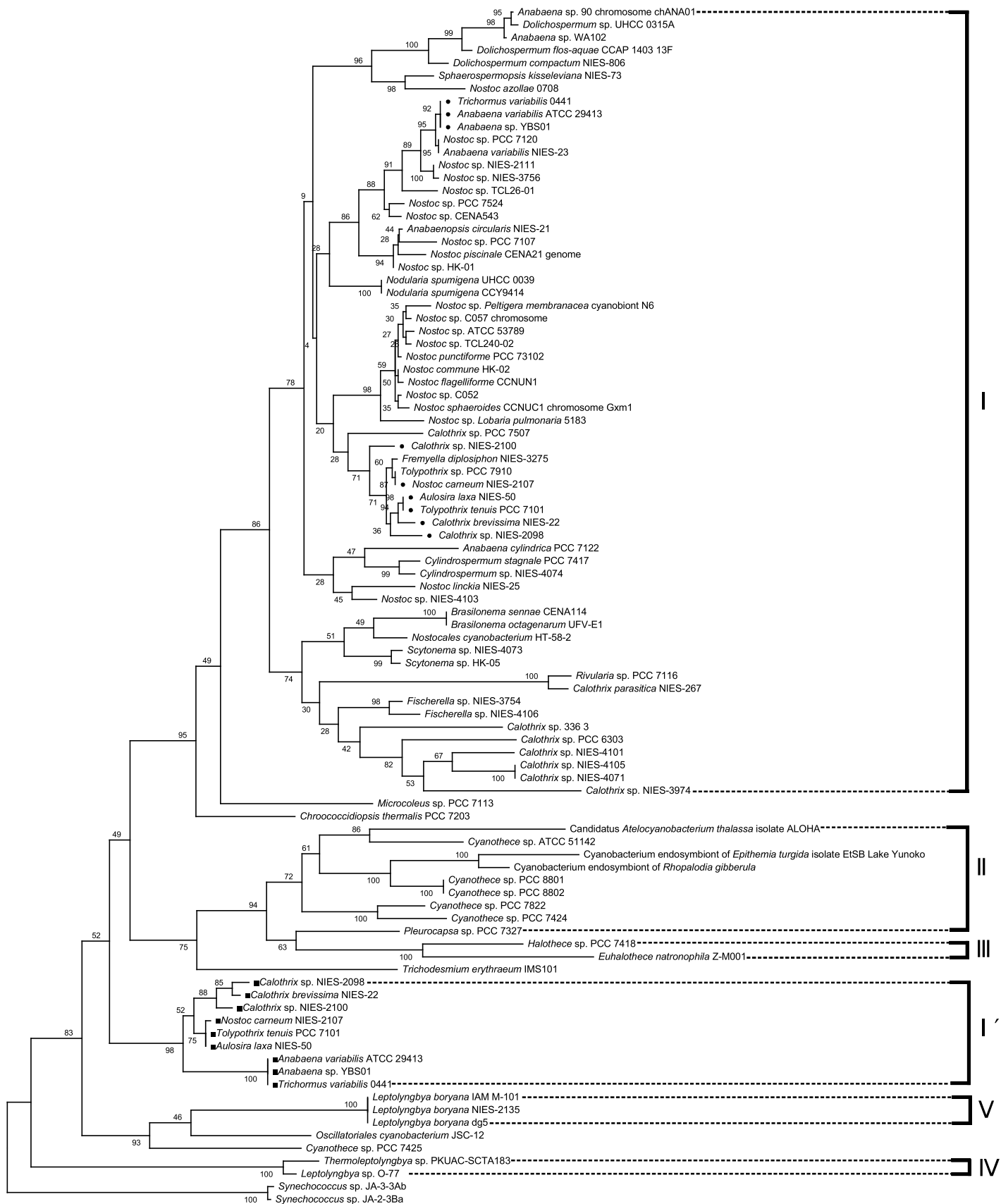

Figure S2b Phylogenetic tree of *nifS*

Supplement: Supplementary file 1 [file biology-10-00329-s001.zip › Figure S2a-2m/FigureS2b.pdf]

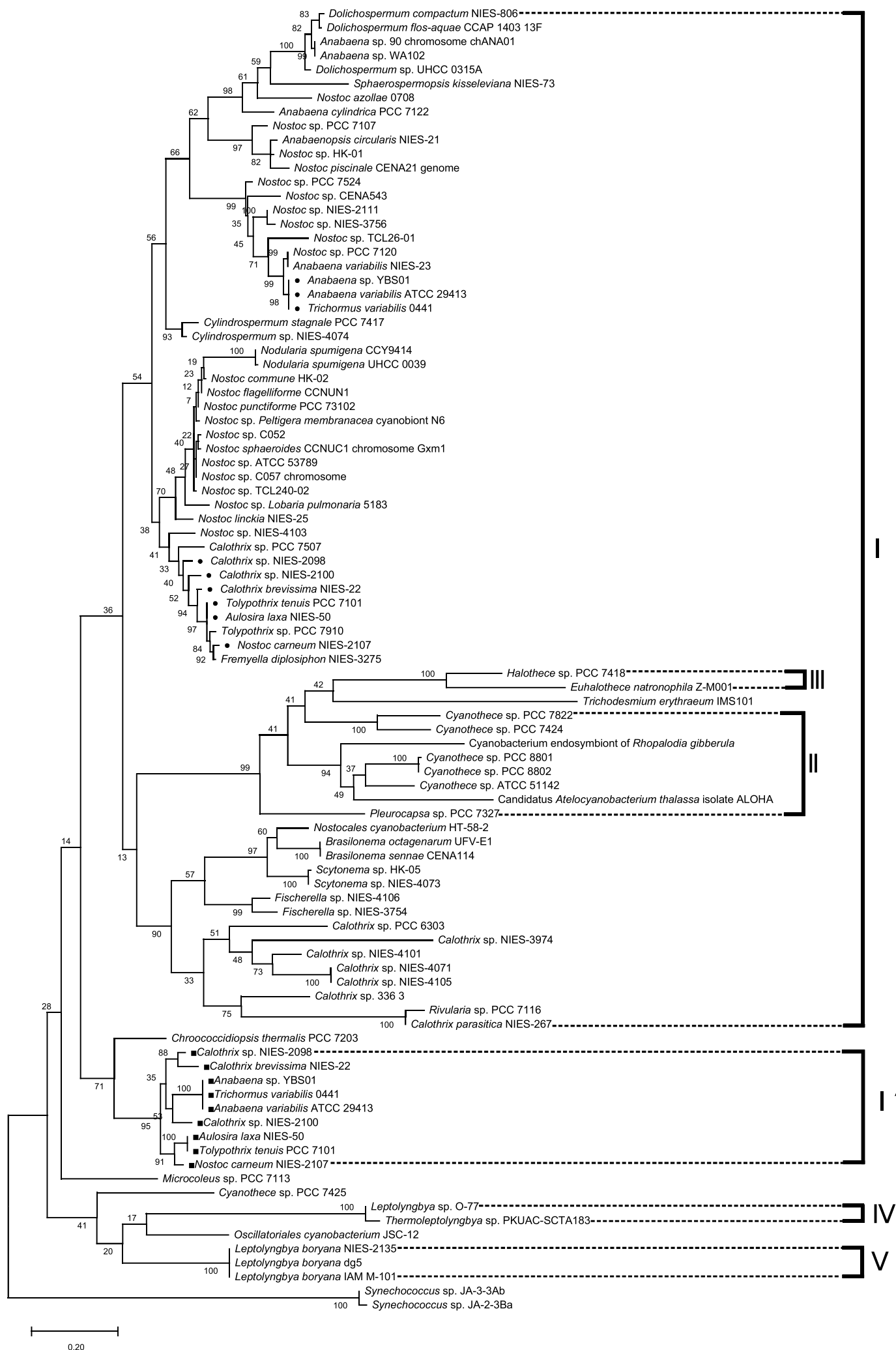

Figure S2c phylogenetic tree of *nifU*

Supplement: Supplementary file 1 [file biology-10-00329-s001.zip › Figure S2a-2m/FigureS2c.pdf]

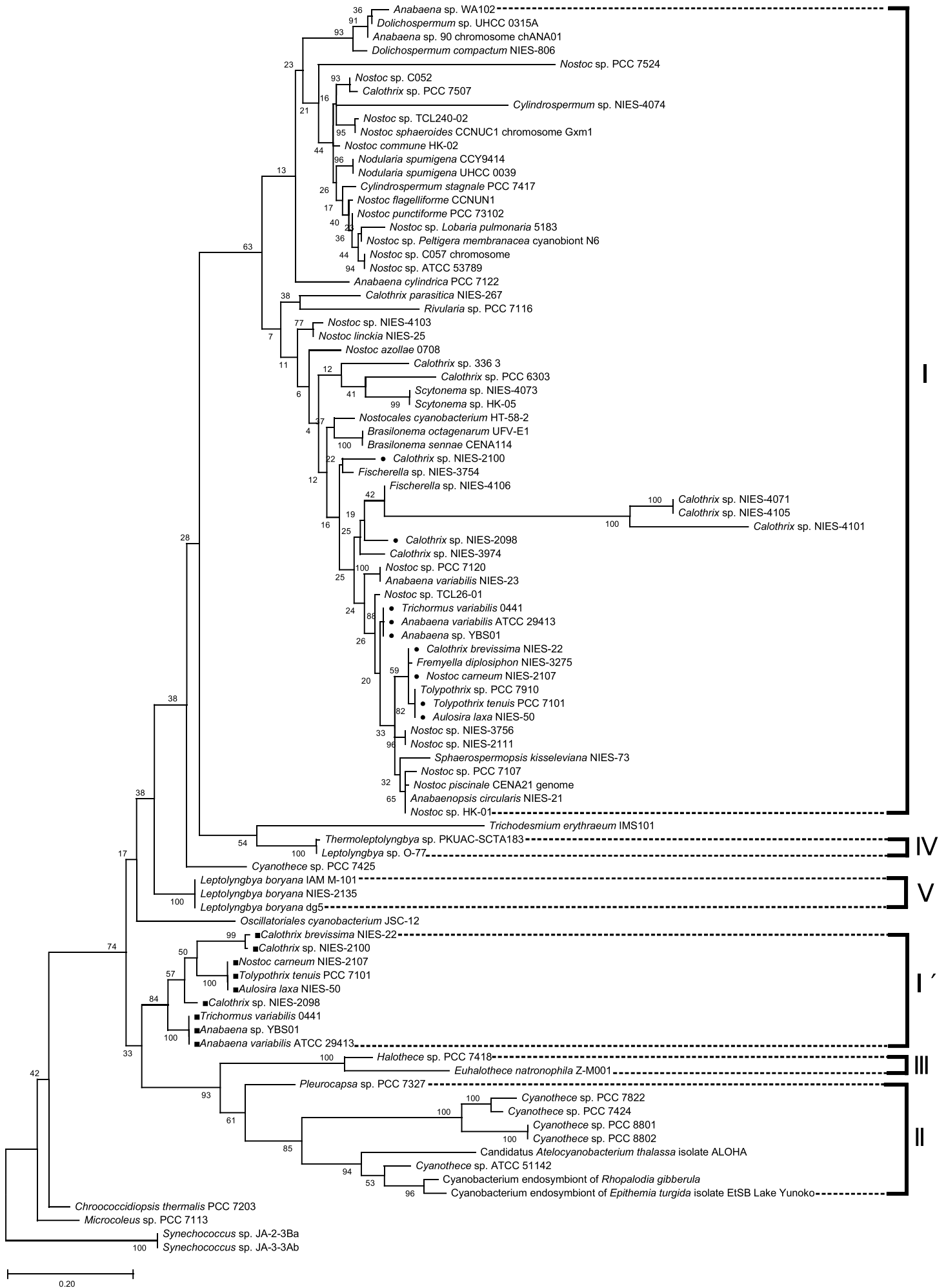

Figure S2d Phylogenetic tree of *nifH*

Supplement: Supplementary file 1 [file biology-10-00329-s001.zip › Figure S2a-2m/FigureS2d.pdf]

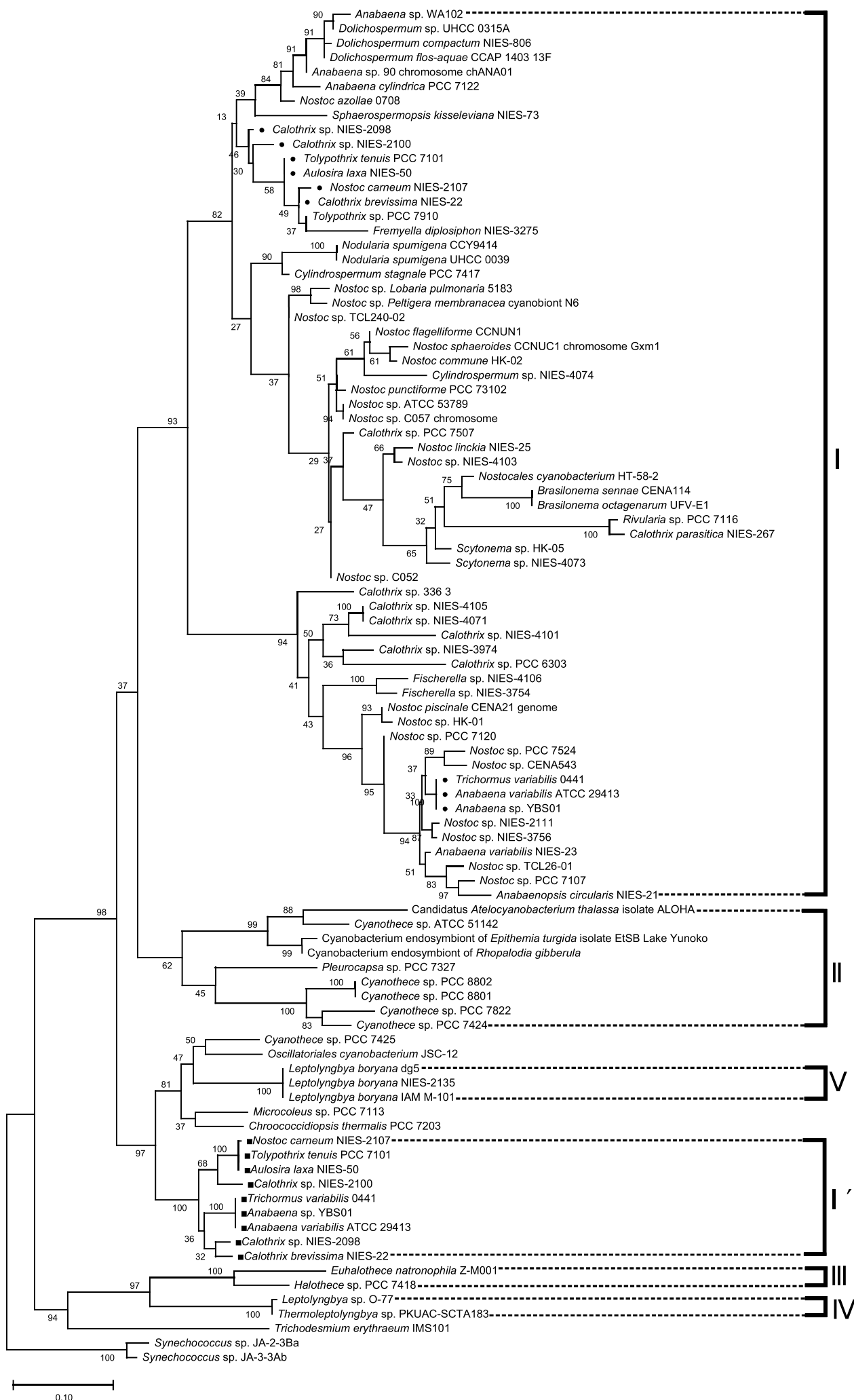

Figure S2e Phylogenetic tree of *nifD*

Supplement: Supplementary file 1 [file biology-10-00329-s001.zip › Figure S2a-2m/FigureS2e.pdf]

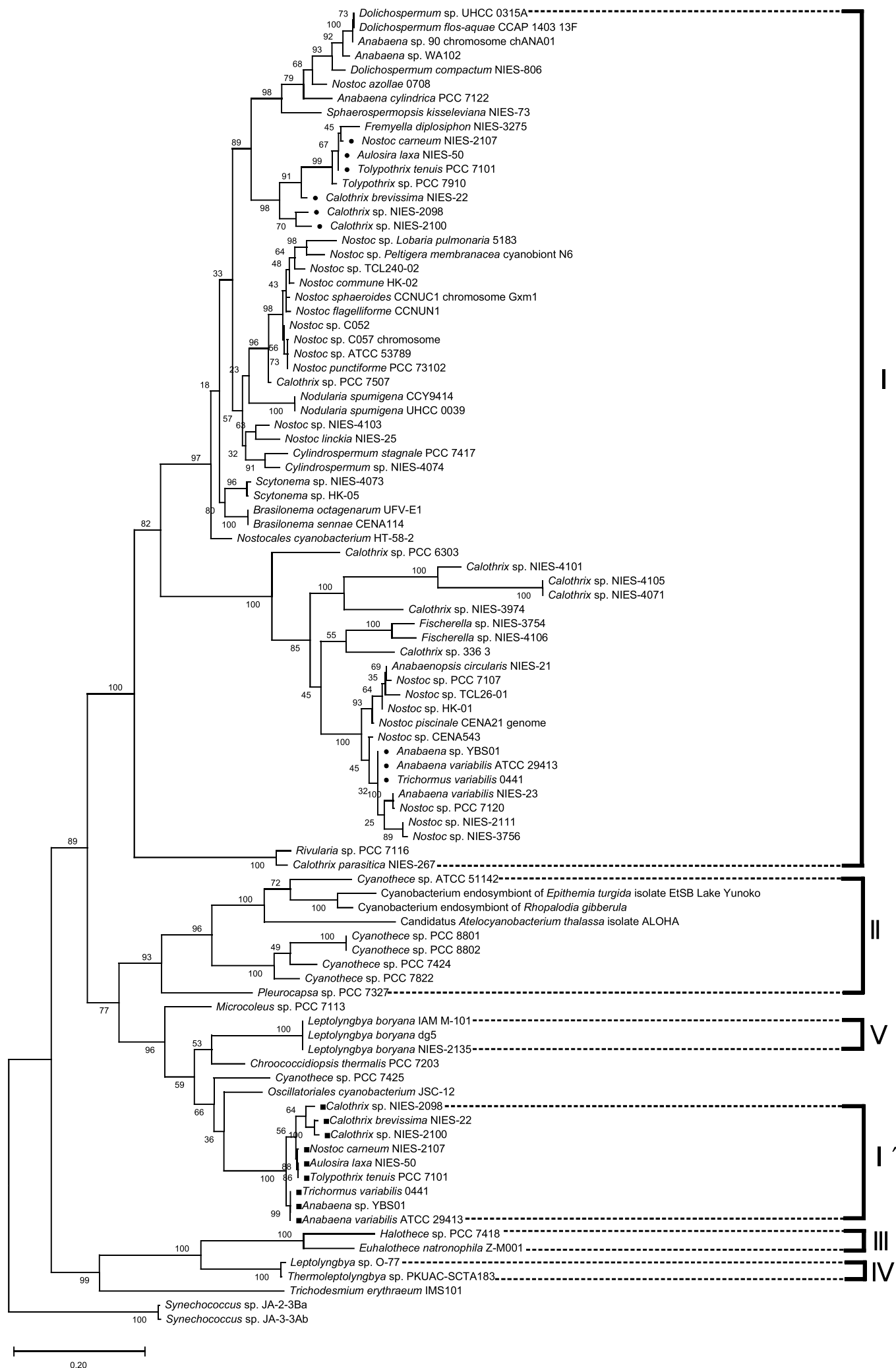

Figure S2f Phylogenetic tree of *nifK*

Supplement: Supplementary file 1 [file biology-10-00329-s001.zip › Figure S2a-2m/FigureS2f.pdf]

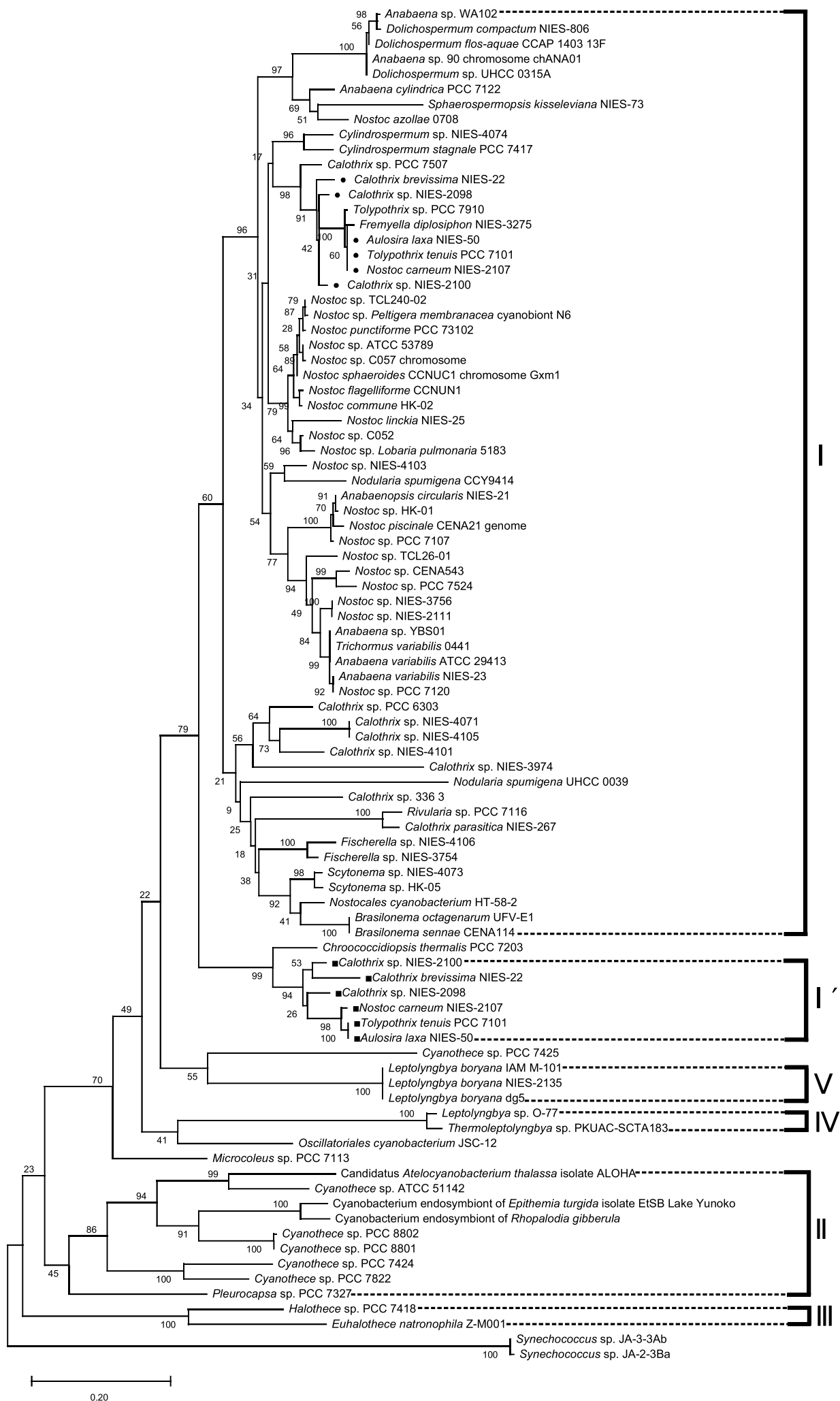

Figure S2g Phylogenetic tree of *nifV*

Supplement: Supplementary file 1 [file biology-10-00329-s001.zip › Figure S2a-2m/FigureS2g.pdf]

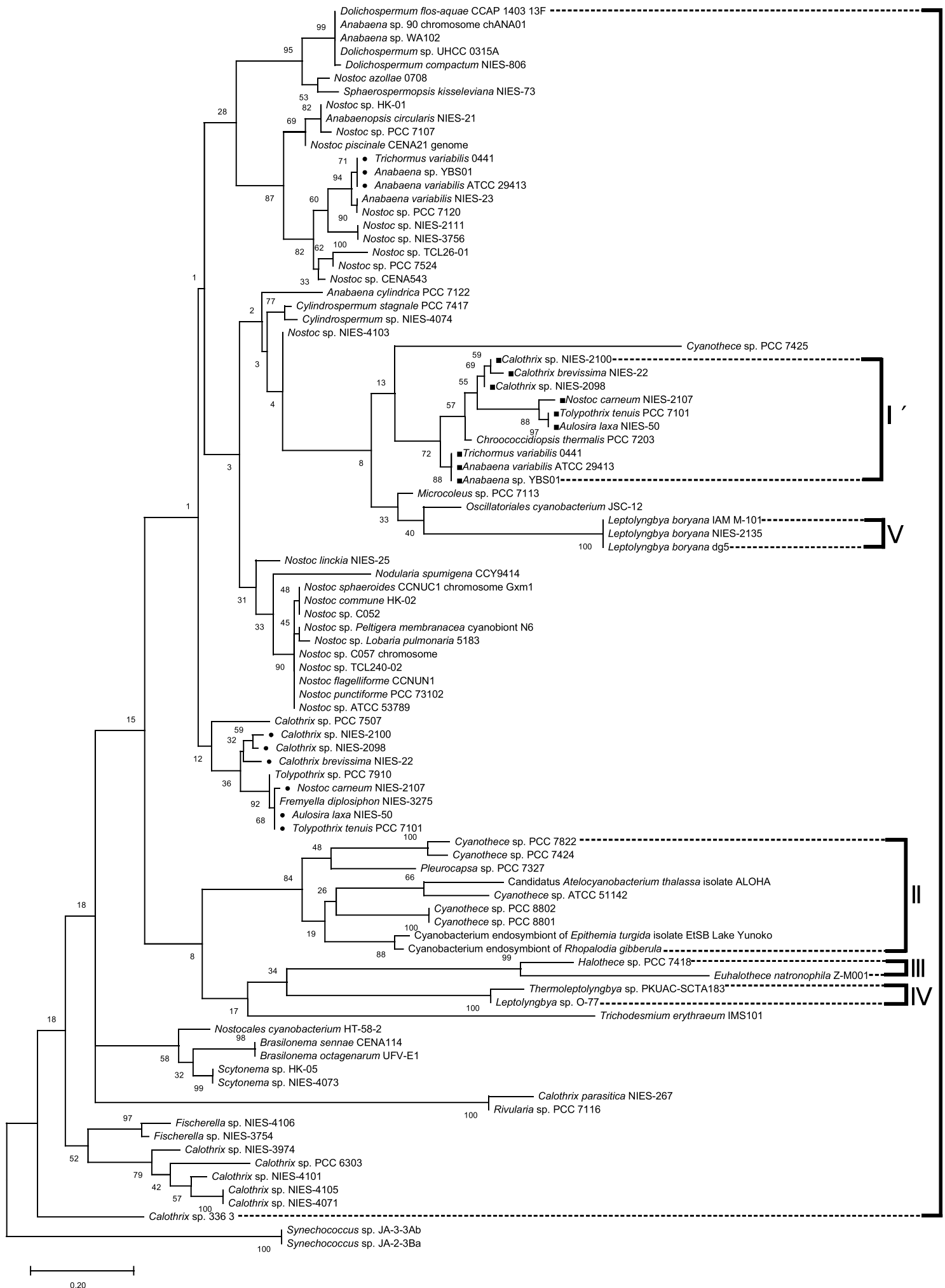

Figure S2h Phylogenetic tree of *nifZ*

Supplement: Supplementary file 1 [file biology-10-00329-s001.zip › Figure S2a-2m/FigureS2h.pdf]

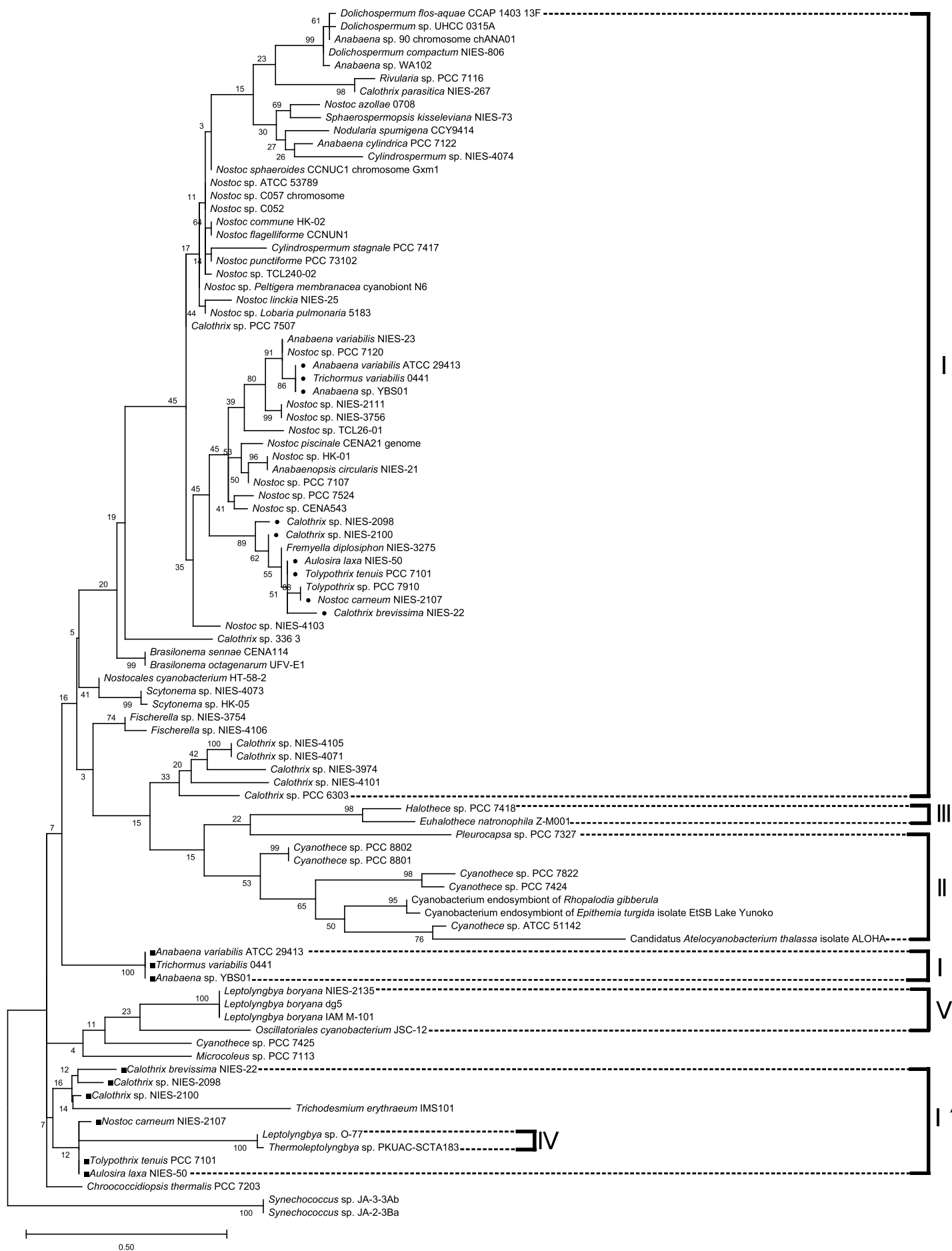

Figure S2i Phylogenetic tree of *nifT*

Supplement: Supplementary file 1 [file biology-10-00329-s001.zip › Figure S2a-2m/FigureS2i.pdf]

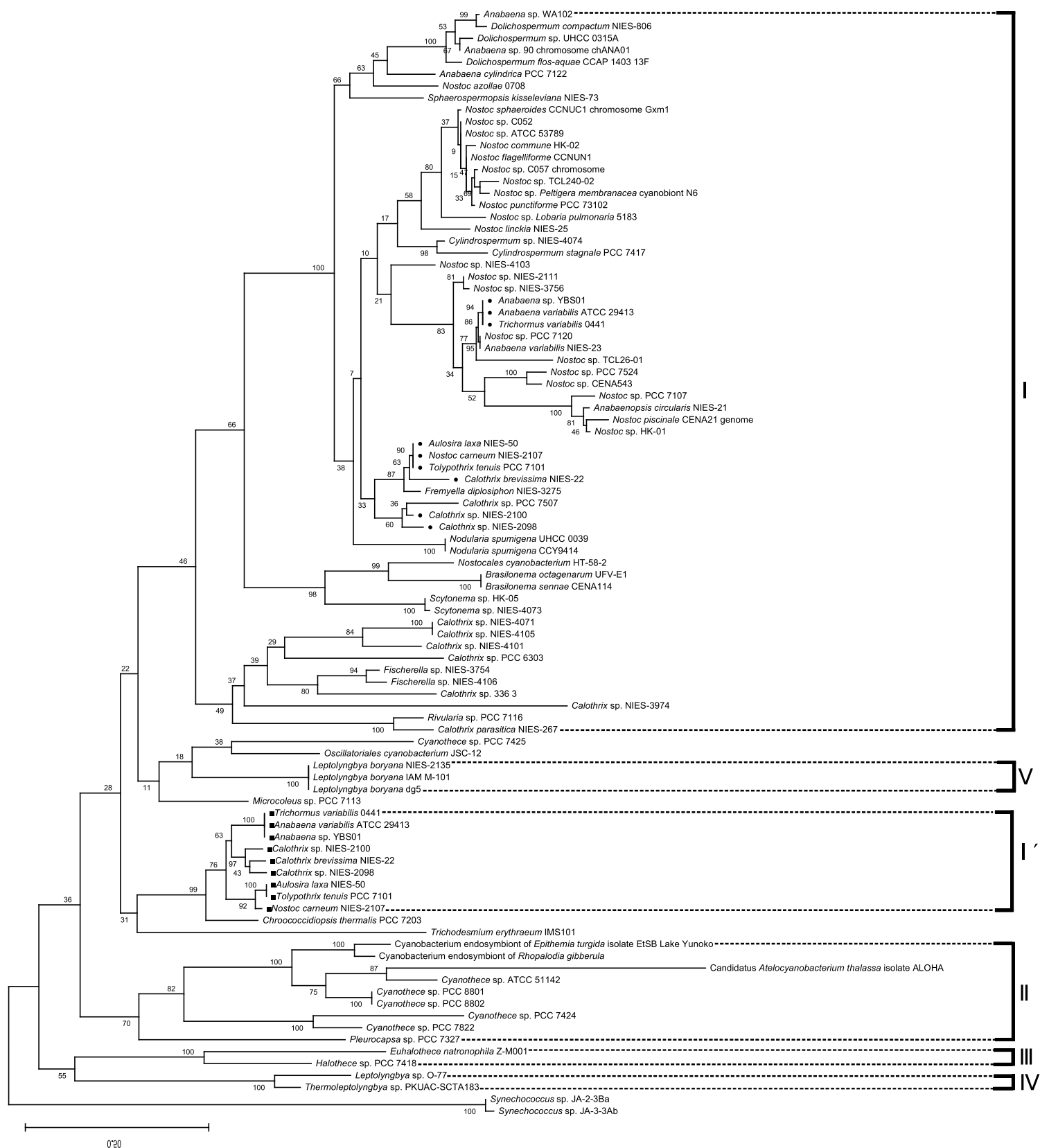

Figure S2j Phylogenetic tree of *nifE*

Supplement: Supplementary file 1 [file biology-10-00329-s001.zip › Figure S2a-2m/FigureS2j.pdf]

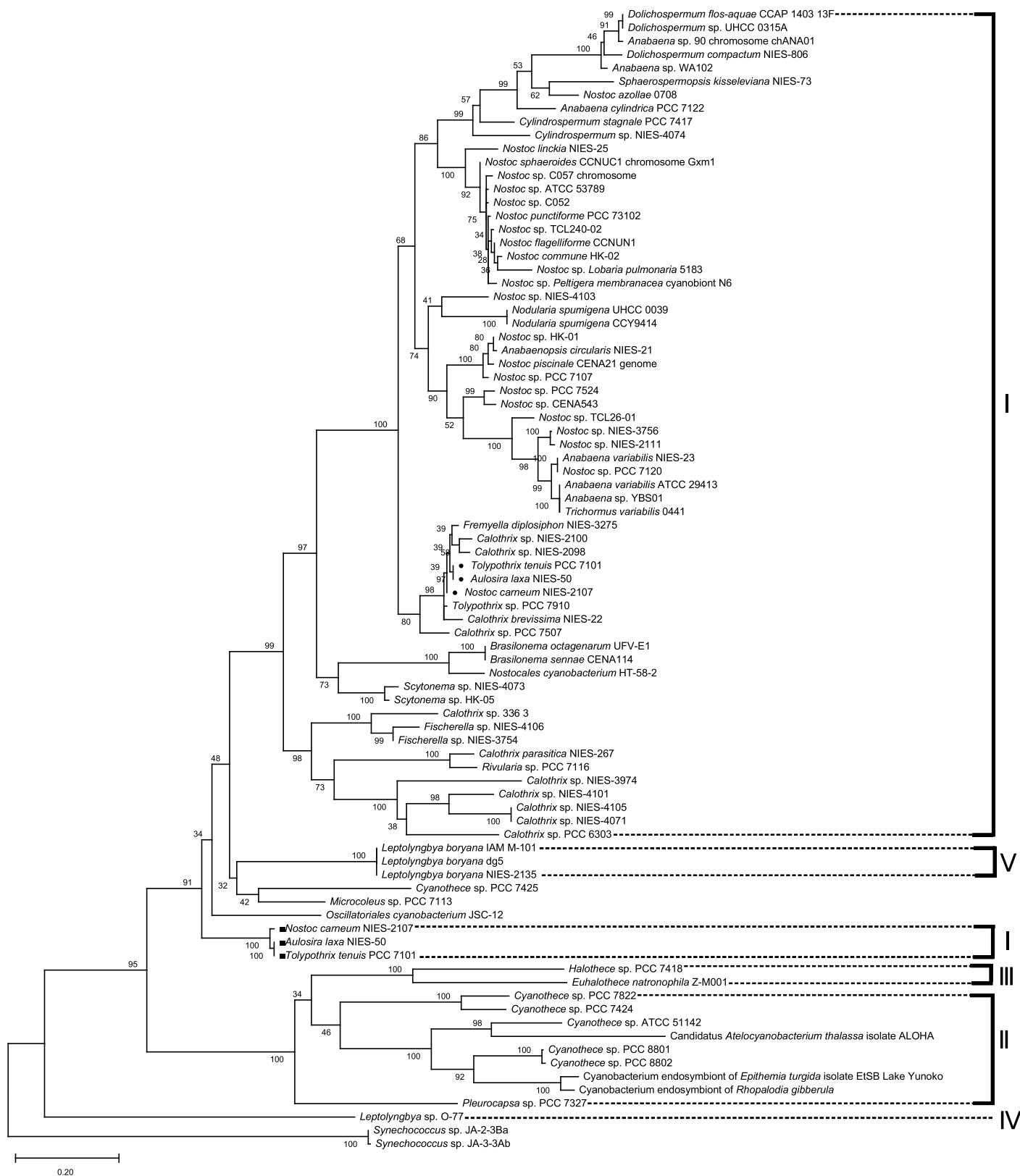

Figure S2k Phylogenetic tree of *nifH*

Supplement: Supplementary file 1 [file biology-10-00329-s001.zip › Figure S2a-2m/FigureS2k.pdf]

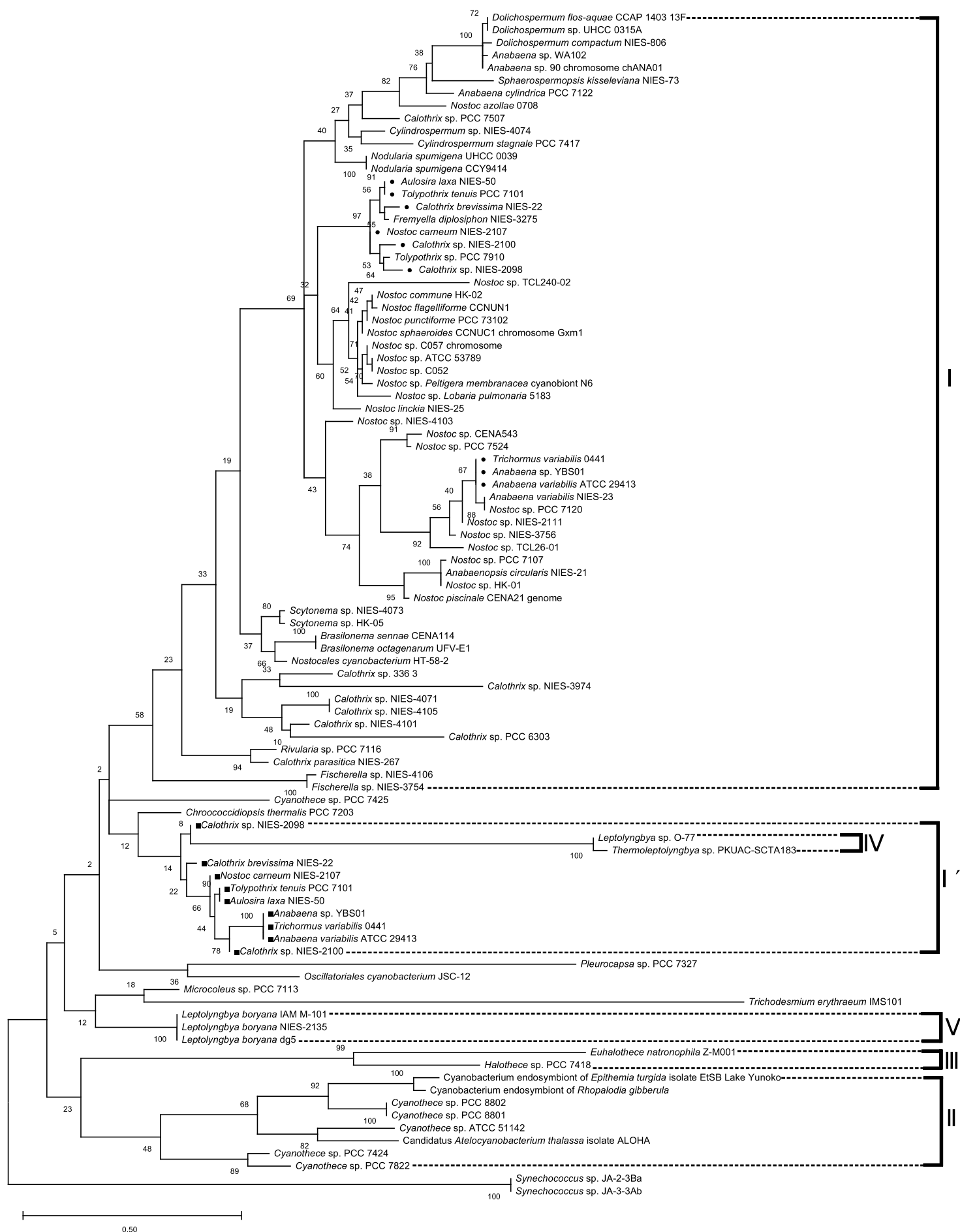

Figure S2l Phylogenetic tree of *nifX*

Supplement: Supplementary file 1 [file biology-10-00329-s001.zip › Figure S2a-2m/FigureS2l.pdf]

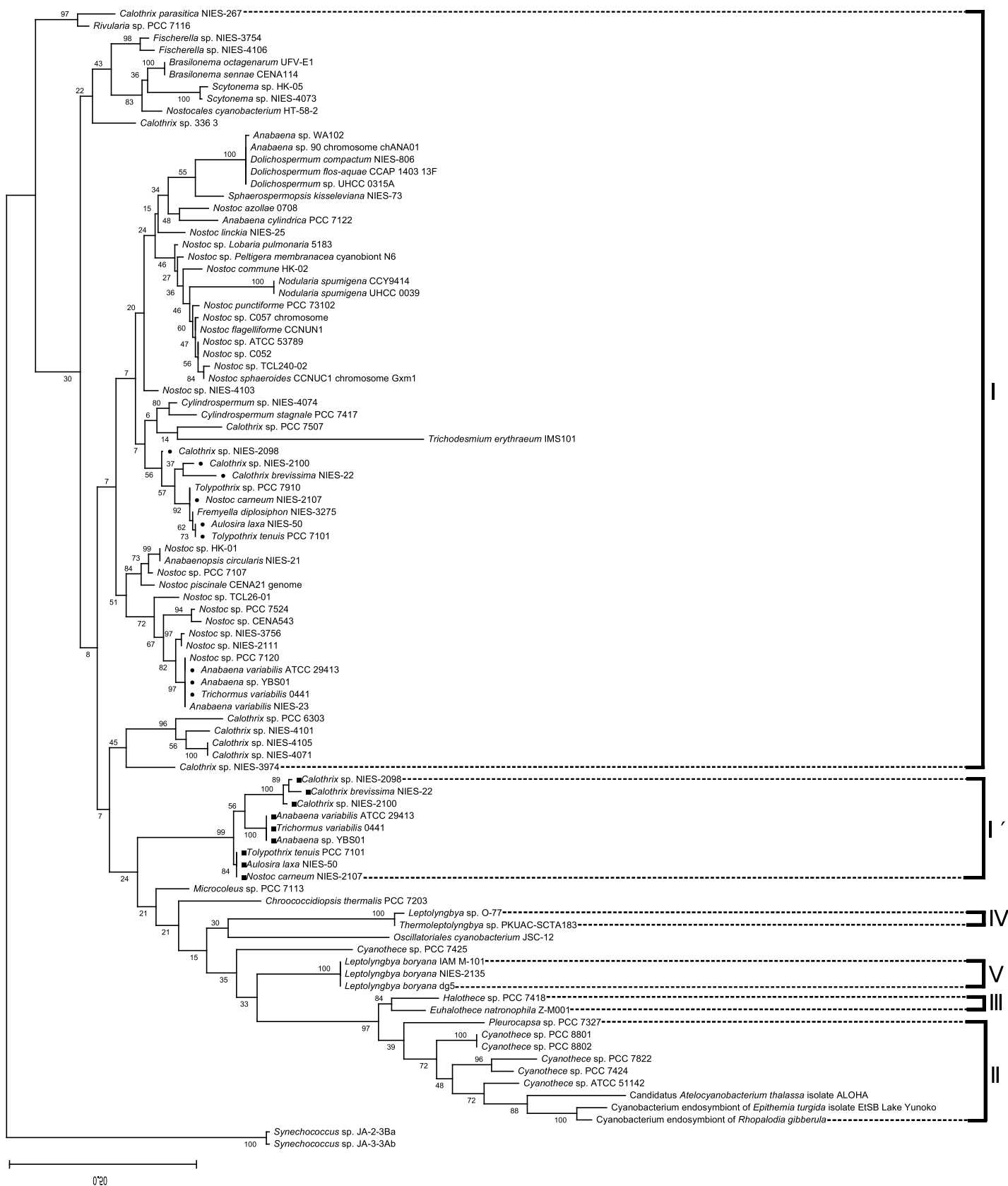

Figure S2m Phylogenetic tree of *nifW*

Supplement: Supplementary file 1 [file biology-10-00329-s001.zip › Figure S2a-2m/FigureS2m.pdf]

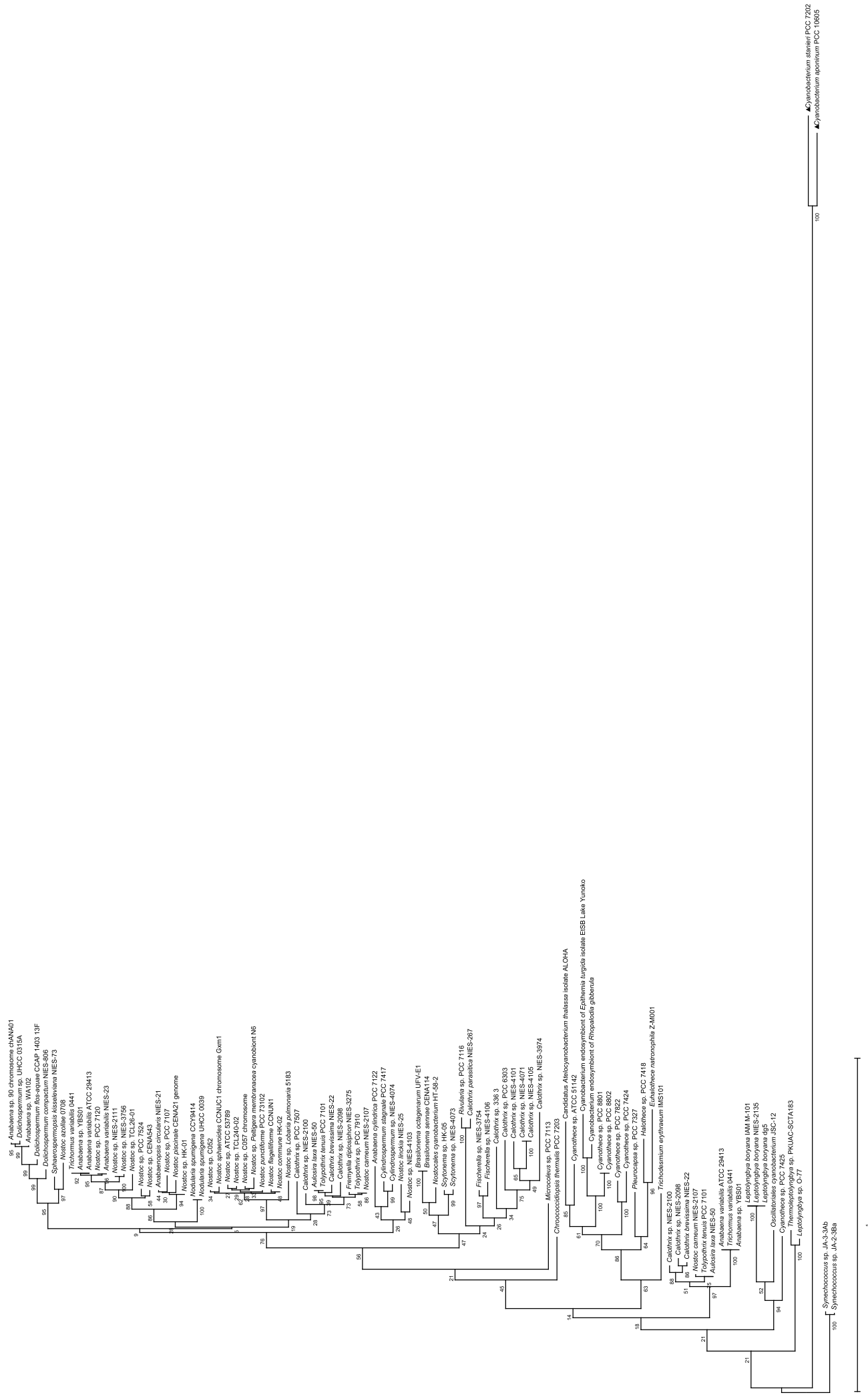

Figure S3a Phylogenetic tree of nifS for diazotrophic and non-diazotrophic Cyanobacteria

Supplement: Supplementary file 1 [file biology-10-00329-s001.zip › Figure S3/FigureS3a.pdf]

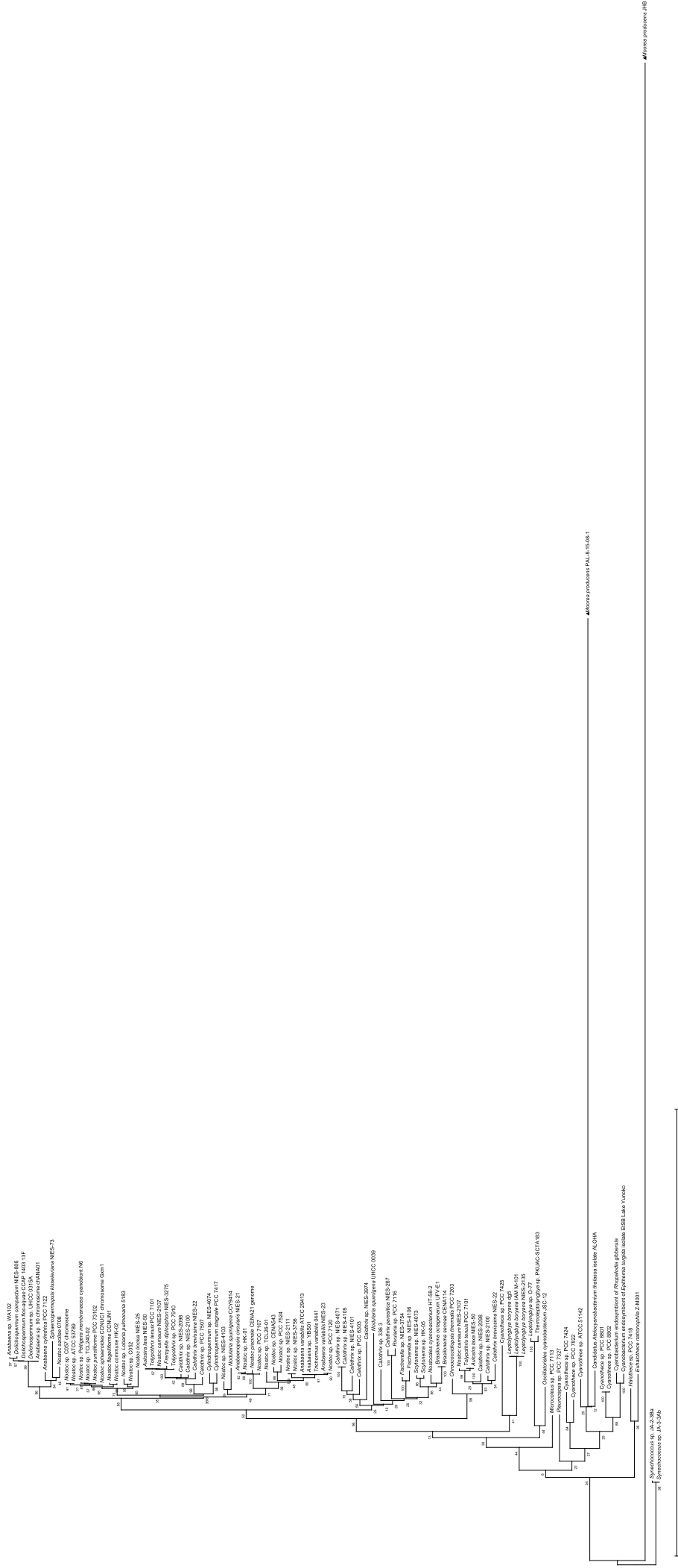

Figure S3b Phylogenetic tree of *nifV* for diazotrophic and non-diazotrophic Cyanobacteria

Supplement: Supplementary file 1 [file biology-10-00329-s001.zip › Figure S3/FigureS3b.pdf]

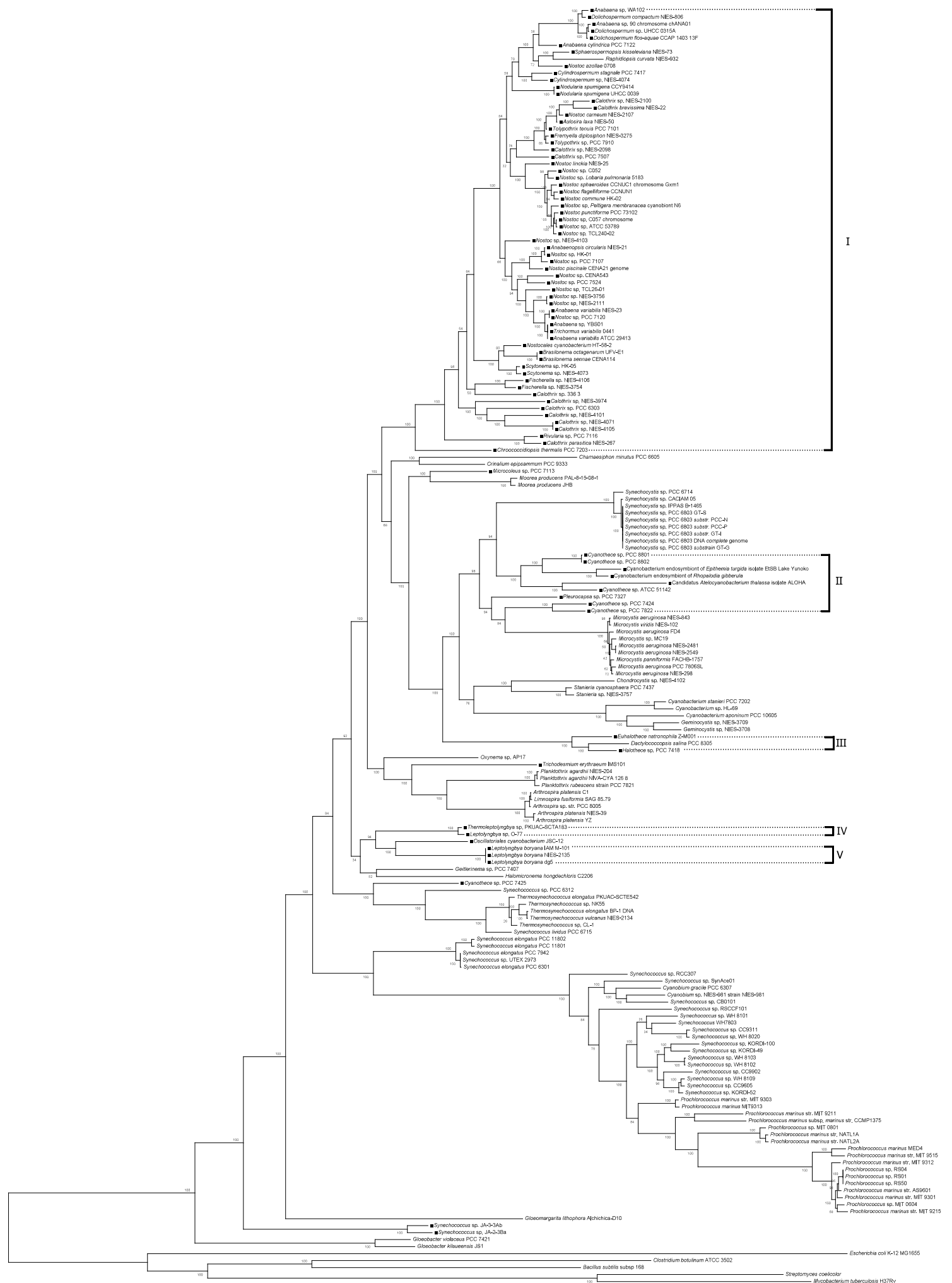

Figure S1 Phylogenetic tree of 179 Cyanobacteria species

Supplement: Supplementary file 1 [file biology-10-00329-s001.zip › FigureS1.pdf]
